# Supplementary material for: PanGraph: scalable bacterial pan-genome graph construction
Source: Microb Genom. 2023 Jun 6;9(6):mgen001034. doi: 10.1099/mgen.0.001034 (PMC10327495; doi:10.1099/mgen.0.001034)
Supplement: Supplementary material 1 [file mgen-9-1034-s001.pdf]

PanGraph: scalable bacterial pan-genome graph construction Supplementary Information

Nicholas Noll,[1,*] Marco Molari,[2,3,*] Liam P. Shaw,[4] and Richard A. Neher[2,3]
[1] *Kavli Institute for Theoretical Physics, University of California, Santa Barbara*
[2] *Swiss Institute of Bioinformatics, Basel, Switzerland*
[3] *Biozentrum, University of Basel, Basel, Switzerland*
[4] *Department of Biology, University of Oxford, Oxford, UK*

I. PANGENOME GRAPH REPRESENTATION

This section contains a short explanation of the pangenome graph representation used in *PanGraph*. A pangenome graph is essentially composed of two elements: pancontigs, which encode the alignment of homologous sequences, and paths, that correspond to representation for genomes as a sequence of pancontigs (see fig. S1).

Each **pancontig** carries the full information on the alignment of homologous sequences it contains. It possesses a single consensus sequence, and each sequence in the alignment is encoded as variations over this consensus. These can be either mutations of single nucleotides, gaps, deletions or insertions. The alignment sequence can be reconstructed by performing these changes in order, as displayed in fig. 2. More information on the precise encoding used can be found in PanGraph documentation.[1]

Each **path** is an oriented collection of pancontigs that represents a genome. For each item in the sequence along with the pancontig id, PanGraph also stores the pancontig orientation (whether homology with the consensus is on the forward the reverse strand of the genome). Since a sequence can traverse a particular pancontig multiple times

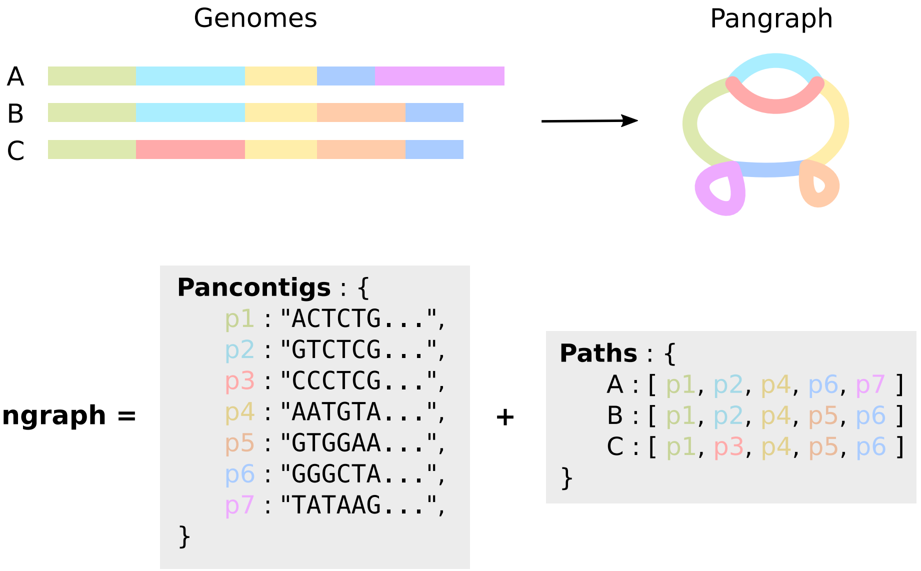

Fig. S 1 **Pangenome graph representation**. Given a set of genomes *PanGraph* will construct a pangenome graph by condensing all homologous sequences in *pancontigs*, containing the alignment of these sequences. Genomes can then be represented as *paths* through the graph, i.e. lists of pancontigs that are traversed when moving along the genome. In our representation a pangenome graph is in essence a collection of *pancontigs* and *paths*.

---

[*] These two authors contributed equally
[1] See `https://neherlab.github.io/pangraph/tutorials/tutorial_2`

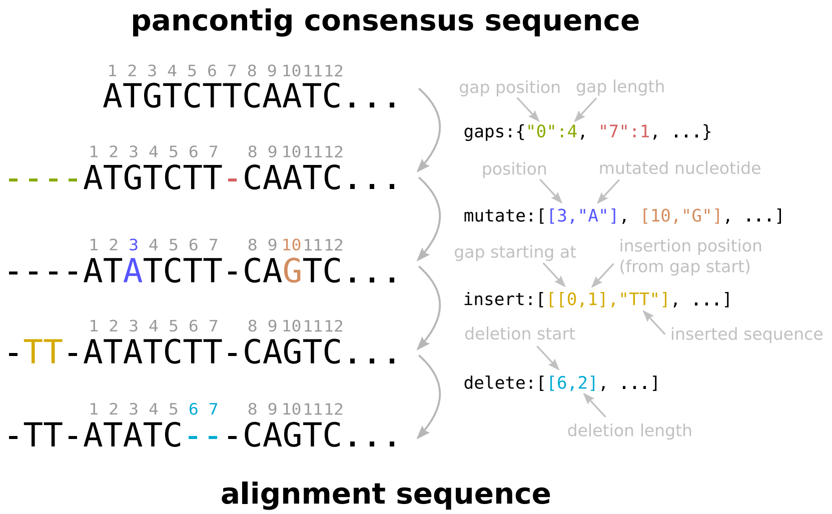

Fig. S 2 **From pancontig consensus to alignment**. In our pancontig representation, each sequence in the alignment is encoded as variations over the pancontig consensus. In practice in the pangraph output `json` file each alignment sequence is saved as a list of gaps to be added to the consensus, sites to be mutated, insertions to be performed in gaps, and deletions. By performing these changes in order on the consensus each sequence in the alignment can be reconstructed. In this example we display how each of these variations are encoded and performed.

(e.g. paralogs), an additional index labels possible repeated occurrences in the pancontig.

From knowledge of all the pancontigs and paths, every single genome can be exactly reconstructed. Every part of the sequence is encoded in a pancontig. "Small-scale" differences between genomes (i.e. SNPs, small insertions and deletions) are encoded in pancontigs as variations over the consensus. "Large-scale" differences, such as presence or absence of long stretch of sequences, are instead encoded in paths as presence or absence of pancontigs. This scale separation can be tuned using PanGraph parameters, as will be explained in following sections.

II. PANGRAPH ALGORITHM

In this section we describe in more detail the algorithmic procedure used to build a pangenome graph. In essence, the full pangenome graph is built by placing genomes on terminal leaves of a guide tree built from a sequence minimizer distance matrix. These genomes are initially considered as elementary graphs composed of a single *pancontig*. These are then propagated along the tree towards the root, and at each intersection a merge operation is performed. The full pangenome graph is then collected from the root.

Below, we discuss the following points in greater detail:

- The estimation of pairwise distances between genomes, used to build the guide tree.
- The procedure used to merge two pangenome graphs together, which is at the core of *PanGraphs*'s algorithm.
- The effect of the main parameters of the algorithm on the resulting graph structure.
- How pangraph processes duplicated regions and possible strategies for paralog splitting.

A. Guide tree construction and distance matrix estimation

As stated in the main text, the guide tree is built by neighbor-joining, with the pairwise distance between sequences being approximated by the Jaccard distance between sequence minimizers. To maximize parallelization, the final tree is a balanced binary tree with the same ordering of leaves as the one found by neighbour-joining. Here we briefly discuss how the complexity of this distance matrix estimation scales with the number n and length L of input sequences.

As a first step of the distance estimation, we sketch all input sequences together and build a unique sorted list of minimizers. This can be done in time $O(\mathcal{L} \log \mathcal{L})$, where $\mathcal{L} = n \times L$ is the total length of input sequences. With each minimizer in the list we also keep the information of the genome of origin.
As a second step we count the number of minimizers shared by each pair of sequences. This can be done efficiently by scrolling the sorted list of minimizers (having size at most proportional to $\mathcal{L}$) only once. For each chunk of equal minimizers in the list (having size between 1 and n, depending on common the original k-mer is) we increment by one the corresponding pairs in a minimizer count matrix. Depending on how similar the input sequences are, this operation has complexity between $O(n^2 L) = O(n\mathcal{L})$ (all minimizers shared) and $O(nL) = O(\mathcal{L})$ (no minimizer shared).
In conclusion, depending on the similarity of the input sequences, evaluation of the pairwise distance matrix has complexity between $O(\mathcal{L} \log \mathcal{L})$ and $O(\mathcal{L} \log \mathcal{L} + n\mathcal{L})$.

In practice for typical use cases of pangraph ($n \sim 100$ and $L \sim 10^6$) the computationally challenging part is the minimizer sketching, and not the distance matrix computation. This is partly due to some prefactors (e.g. the window size of 100 bp used for sketching makes so that the list of minimizer is significantly shorter than the genome, and the only operation performed n^2 times is a single increment of an integer in the minimizer hit matrix). Moreover, the computational time to build the guide tree is usually negligible compared to the time required for the graph merging procedure.

B. Graph merging

At each merge operation two pangenome graphs need to be joined into a single one. Three of *PanGraph*'s parameters play a role in the outcome of this procedure:

- the minimal pancontig length $L_{\min}$.
- pseudo-energy parameters α and β (cf. eq. (1) in the main text).
- the choice of alignment kernel (minimap2 or mmseqs2) and aligner precision (asm5, asm10 or asm20 for minimap2).

In the merging, these parameters control how much of the diversity between sequences must be stored as "large-scale" variation (difference in paths) and how much as "fine-grained" variation (differences in alignments, stored in pancontigs). This is done for example by setting a divergence limit for sequences to be stored in the same alignment.
An implicit assumption of our procedure is that the phylogeny of sequences stored inside the same pancontig is roughly star-like, with each sequence containing independent variations from the consensus. In such cases the consensus is a good "average" representation for the set of sequences in the alignment. In the next section we briefly discussion how PanGraph behaves when this assumption is not satisfied, such as in the case of merging paralogs in the same pancontig.

The merging operation is performed in the following steps:

1. Using the chosen aligner kernel, we scan for homology between pancontigs of the two different graphs. This is done by using only the consensus sequence of each pancontig.
2. The aligner will return a set of matches. Each match is characterized by an alignment length and a measure of diversity. Using this information we rank matches by pseudo-energy, so that longer and less diverged matches will be processed first. We refuse all matches with positive pseudo-energy and all matches shorter than the threshold length $L_{\min}$.
3. For each of the remaining matches we attempt to merge pancontigs following these steps:
   (a) We check that no prior mergers have been performed on the same two pancontigs. Sometimes multiple matches are found on the same pancontig, and in this case we prioritize the ones with more negative pseudo-energy. Any following match on the same pancontig will be discarded at this step. It will however be performed later on in the self-map phase of the algorithm (see step 4).
   (b) Flanking regions of the consensus sequences that are not part of the match are cut and not merged and stored in separate pancontigs (see fig. 1 right panel in the main text). One exceptions is when any of these flanking regions is shorter than the threshold length $L_{\min}$. In this case instead of creating a very short pancontig the region is stored as an indel in the merged pancontig alignment.

(c) The remaining homologous region is then a candidate for the merging. However the merging might result in more than a single pancontig, depending on the underlying alignment. If the alignment contains insertions or deletions longer that $L_{\min}$, these need to appear as separate pancontigs. To this end the CIGAR string is parsed, and the long match is potentially split in multiple sub-matches, with interposed long insertions or deletions that will be saved as separate pancontigs.

(d) At this point every (sub-)match is processed to produce a single merged pancontig. This is done by expanding the alignments of the two original pancontigs, and stitching them together following the instructions contained in the CIGAR string of the match. Once the full alignment is constructed the new consensus sequence is re-evaluated, and each sequence is then encoded as variations over this consensus. This completes the creation of a new pancontig.

(e) The original starting pancontigs are then removed from the pangenome graph, and no new mergers can be performed on them at this step of the algorithm. The newly-created pancontigs are added to the graph and the corresponding paths are updated.

4. Once the list of all matches has been exhausted the two graphs have been merged into one. However at the previous stage only one merger could be performed per pancontig, which could be insufficient to collapse all of the homology contained in the graph. To this end we repeat the procedure described in step 3, but instead of looking for matches between pancontigs of two different graphs, we perform an all-against-all homology search between all pancontigs in the same graph. Mergers are performed following the same rules, and this procedure is repeated until no new mergers can be performed.

5. In the final step of the procedure we perform some final grooming of the graph. This includes for example removing transitive edges, i.e. pairs of pancontigs that are always traversed in the same way by all paths. These pairs are merged in a single pancontig. This can happen for example when dealing with circular sequences such as bacterial chromosomes. These sequences are transformed into a single linear pancontig at the beginning of the algorithm, which introduces an artificial cut. When this elementary graph is propagated and the unique pancontig is split, a transitive edge is generated across the artificial cut. We remove it in this step.

As graphs are propagated along the guide tree more and more mergers are performed. This makes pancontigs progressively shorter but their alignments deeper.

During the graph merging procedure, only consensus sequences from each graph are used to find homologies between pancontigs, not the individual sequences represented by the pancontig. This makes this procedure fast and is sufficient to capture the large-scale structural variation of the pangenome, but might introduce minor inconsistencies and artifacts in the alignment. To this end *PanGraph* has the command `polish`[2]. This command "polishes" alignments by aligning all sequence represented by the pancontig using MAFFT (Katoh *et al.*, 2002). The subcommand has a simple interface: it takes as input a pangenome graph and produces another pangenome graph with the same structure but improved pancontig alignments. This efficiently separates the problem of finding large-scale structural variation, and nucleotide-level variation by using established algoritms for multiple sequence alignment only on parts of the input genomes with established homology.

C. PanGraph parameters

After explaining the procedure we use for merging, we comment briefly on the effect of *PanGraph*'s parameters on the resulting pangenome graphs.

Parameter $L_{\min}$ controls the minimal size of pancontigs. Indels shorter than this size are saved as variation in alignments, rather than as separate pancontigs. As such this parameter partially controls the fragmentation of the graph. The appropriate value depends on the scale at which one wants to study structural variations in genomes. The default value in PanGraph is set to 100 bp. This is done to capture structural variation at the gene scale, while ignoring small indels. The value can, however, be tuned by the user depending on the specific use-case.
A similar role is played by the parameter α. This can be interpreted as the cost of introducing a cut in a pancontig.

[2] See `https://neherlab.github.io/pangraph/tutorials/tutorial_3/`.

The length of homologous sequence to be merged must exceed this cost for a cut to be performed.

The choice of alignment kernel and the value of β set instead a limit on the divergence of sequences that are merged in the same pancontig. The first intrinsic limit is set by the choice of alignment kernel and its sensitivity: minimap2 is significantly faster than mmseqs2, but it is in general unable to find matches between homologous sequences with more than 20% divergence (with asm20 sensitivity).
On top of this intrinsic limit of the aligner, more explicit control of the acceptable amount of divergence can be achieved by tuning the value of β. From the definition of the pseudo-energy (cf. eq. (1) in the main text) it follows that matches between homologous sequence with divergence higher than $1/\beta$ will have positive pseudo-energy and will thus not result in a merger. If the molecular clock hypothesis holds, tuning this parameter is equivalent to setting an effective time limit beyond which contigs are not to be merged.

D. Paralog splitting

By design, in *PanGraph* duplicated sequences (of size larger than L_{min}) will be merged in the same pancontig. This is done by design, so that positions in which these duplications are present will correspond to a structural breakpoint in the graph, and information on all of the contexts in which a duplicated region appears will be grouped together in the edges connected to this duplicated pancontig.
This is however not always desirable. For example in the case of distantly related paralogs the user might want to avoid unnecessary structural complication in the graph, and prevent merging these sequences together if they are generated by an ancient duplication event.

This paralog splitting can be done using two different sources of information: either sequence diversity or local context in which the duplication is inserted. Currently *PanGraph* does not provide a specific command to perform paralog splitting. This is a feature that we plan on implementing in the future. However partial control of paralog split can currently be achieved in two ways.
The first one is based on sequence divergence. By tuning the value of the energy parameter β the user can set a divergence limit. This would prevent the merging of paralogs that have sequence divergence higher than $1/\beta$ during graph construction.
The second method relies instead on context. The `marginalize` command possesses the optional `--reduce-paralog` flag. When set, this flag will result in the removal of artificial splits caused by duplicated pancontigs that always occur in the same non-duplicated context. These duplicated pancontigs are then split and absorbed in their context, thus simplifying the graph and loosing trace of the duplication.

III. GENERATION OF SYNTHETIC DATA

As a first test we benchmark the performances and accuracy of *PanGraph* on synthetic data, obtained by simulating the evolution of a population in which individuals accumulate mutations, can loose part of the sequence from deletions, or gain new genetic material via Horizontal Sequence Transfer (HST) from other members of the population.
In the simulation a population of size N is evolved for T generations using a Wright-Fisher model (Hudson, 2002). Each individual in the population has a circular genome of initial size L. At each generation single-nucleotide mutations occur at a rate μ per position. Deletions can occur at a rate d per genome per generation. When a deletion is suggested on isolate n, having a genome length L_n, a new desired length L'_n is extracted from a Gaussian distribution with mean L and variance σ^2. If $\Delta = L'_n - L_n < 0$ then a random chunk of length $|\Delta|$ is removed from the genome, reducing it to size L'_n. If $\Delta > 0$ no deletion is performed. This ensures that the length distribution of genomes in the population remains close to the desired length L, with σ^2 being a proxy for the variance. HST occurs at a rate h per genome per generation. Similarly to deletions, when a HST event is suggested a new desired length L'_n is extracted from the same Gaussian distribution, and this time the event is performed only if $\Delta = L'_n - L_n > 0$. In this case a random chunk of sequence of length Δ is extracted from a random individual in the parent population (possibly the parent of isolate n itself) and inserted in a random position of the genome of isolate n. All parts of the sequence have equal probability of being transferred, irrespective of homology. Finally, inversions occur at a rate i per genome per generation. When an inversion is proposed a random length Δ is chosen in the same way, and a random chunk of sequence of this length is inverted. In the simulation we do not implement a notion of genes. Standard values of the

simulation parameters are reported in Table I.

Parameter	Description	Standard Value
N	population size	100
T	n. of simulated generations	50
L	average genome size	50 000 [bp]
σ	s.t.d. of genome size	$L/10$ [bp]
μ	mutation rate	0.005 [1/bp · generation]
d	deletion rate	0.05 [1/generation]
i	inversion rate	0.01 [1/generation]
h	HST rate	0.1 [1/generation]

TABLE I **Simulation parameters**. Description of all simulation parameters used for the generation of synthetic data. Unless otherwise specified, the standard value is used.

At the end of the simulation the resulting mosaic genomes are collected, along with the real underlying pangenome graph that can be used as the ground truth against which to check the performances of *PanGraph*. The code to perform these simulations is shipped with *PanGraph* in the `generate` command.

IV. BENCHMARK OF PANGRAPH ON SYNTHETIC DATA

We test the performance of *PanGraph* on synthetic data, focusing on two aspects: the computational performances of the algorithm (time and memory requirements) as a function of the dataset size, and the accuracy of the algorithm in reconstructing the real pangenome graph of the population as a function of sequence divergence.

A. Computational performance

To test the computational performances of *PanGraph* we generated data using the model described in the previous section, with standard value of the parameters but varying the average genome length $L = [1, 5, 10, 50, 100, 500]$ kbp and the population size $N = [10, 20, 50, 100, 200, 500, 1000]$. For each pair of N, L values we generated 50 different datasets. On each dataset we ran the *PanGraph* `build` command, using *minimap2* as alignment kernel with *asm20* option. We used standard value for the energy parameters $\alpha = 100$, $\beta = 10$. Runs were performed using 8 cores. For each run we measured the wall-time of the command, the maximum memory requirements (maximum resident size) and the average cpu percent. Results are displayed in Fig. 3. Thanks to the guide tree architecture of *PanGraph* the run time scales almost linearly with the number of isolates.

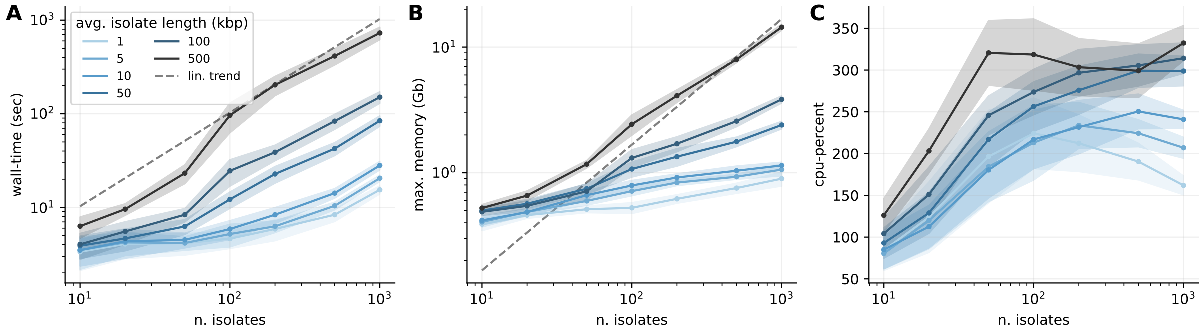

Fig. S 3 **Computational performance of PanGraph algorithm on synthetic data, as a function of dataset size**. We run PanGraph on artificially generated datasets consisting of a variable number of isolates with genomes of variable length (colorscale). For each condition we display the average and standard deviation over 50 different runs of: (A) algorithm wall-time, (B) maximum memory requirement and (C) average percentage of cpu used on a total of 8 cores.

B. Accuracy in reconstructing the pangenome graph

To test the accuracy of *PanGraph* in reconstructing the pangenome graph of a set of isolates we generated artificial data using the procedure described in Section III. For each simulation we obtain both a set of genome sequences and the underlying real pangenome graph. This was done using standard value of the parameters, but varying the rate of HGT in the interval $h = [0.01, 1]$ and the mutation rate $\mu = [0, 0.01]$. For each (h, μ) pair 25 different sets of data were generated.
On each set of data we executed *PanGraph* with values for the energy parameters $\alpha = 0$ and $\beta = 0$, and with three different alignment kernels:

- *minimap2* with option *asm10*,
- *minimap2* with option *asm20*,
- *mmseqs2*,

thus obtaining three different pangenome graphs per dataset.
To link the mutation rate parameter μ with the average pairwise SNPs distance $\langle d\rangle$ between homologous segments in the population we evaluated the average pairwise distance for every pancontig in the pangenome graph with depth greater than one. For each graph we then perform the weighted average of these divergences, using as weight the length of the pancontig. Finally, for every pair of parameters (μ, h) we average these numbers over the 25 different simulations. Results are displayed in Fig. 4.
For high values of μ, the average pairwise divergence of pancontigs is not influenced by the rate of HGT h, but depends strongly on the choice of alignment kernel. With the *asm20* option, *minimap2* is able to correctly merge genomes with average pairwise divergence $\langle d\rangle \sim 7\%$ in our simulations, while *mmseqs2* reaches $\langle d\rangle \sim 10\%$ at the expense of higher processing time. Notice that these values are lower than the threshold sensitivity of these aligners, which are expected to find matches up to respectively around 10%, 20% and 30% sequence divergence. This is due to the fact that homologous sequences in our simulations are generated by an evolutionary process, and they can have a wide distribution of diversities and hierarchical population structure. Inability to merge sequences above a threshold divergence in a single pancontig is expected to result in multiple pancontigs containing sub-clades with higher similarity, and average diversity that is appreciably lower than the aligner threshold.
By performing a linear fit on the datapoints with $\mu \leq 0.002$ we are able to recover the conversion factor between the mutation rate and average pairwise divergence of pancontigs in our simulations: $\langle d\rangle \sim 20.8\,\mu$.

Once this link has been established we group simulations by the value of h and measure accuracy by comparing the reconstructed pangenome graphs with the ground truth provided by our simulations. In particular, for each isolate we consider the breakpoints between different pancontigs that tile the genome, and measure the displacement between the real position of these breakpoints and the position reconstructed by pangraph.
To evaluate this displacement it is first necessary to link breakpoints on the real and reconstructed pangenome graph. We formulate this problem as an assignment problem, and solve it numerically using the Hungarian method (Kuhn, 1955). For each isolate we call b_i for $i = 1, \ldots, I$ the pancontig breakpoints on the real pangenome graph, and b_j for $j = 1, \ldots, J$ the ones on the reconstructed pangenome graph.[3] We define a cost matrix $D_{ij} = d(b_i, b_j)$ whose elements are distances on the genome of pairs of breakpoints from the two graphs. We then numerically solve the assignment problem on this matrix, and obtain a set of $K = \min\{I, J\}$ pairings between breakpoints such that the distance between the pairs is minimal and all the breakpoints of the graph with minimum number of breakpoints have been paired. The average distance between breakpoints in these pairs is the average breakpoint distance of the graph.
For each simulation we obtain a number of average breakpoint distances equal to the number of isolates in the simulation. In Fig. 5 we plot the cumulative distribution of these distances, with a cutoff at 1kbp. We stratify simulations according to the average pairwise divergence of pancontigs inferred from the value of μ. Each panel corresponds to a different alignment kernel. As the sequence diversity increases we observe a clear transition. For low diversity most of the breakpoints are inferred to be only a few bp away from their real position, while for highly diverged sequences the position of breakpoints is not precisely inferred and can be hundreds of bp away from their real position. For different alignment kernels this transition occurs at different values of the divergence, with *mmseqs2* being the most accurate, as can be visualize from Fig. 3 in the main text.

---

[3] To avoid artifacts generated by the default threshold distance $d = 100$ bp of *PanGraph*, if any pair of breakpoints on the same graph sits at a distance smaller than this threshold, we remove one of them.

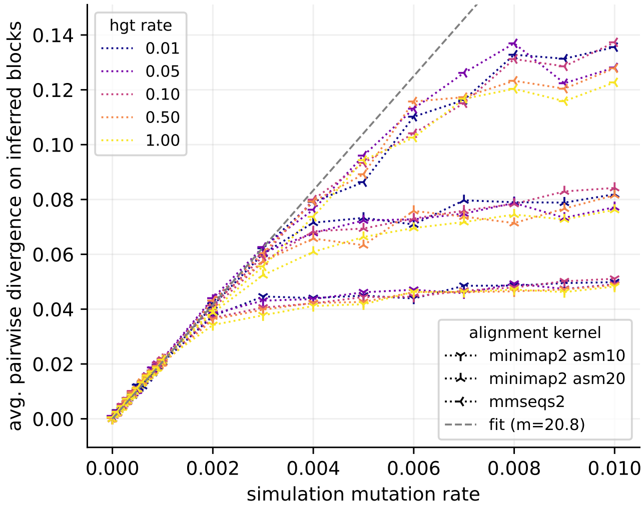

Fig. S 4 **mutation rate vs divergence on synthetic data**. We compare the mutation rate μ of our simulations with the average pairwise divergence $\langle d \rangle$ of sequences in pancontigs of the resulting pangenome graphs. This is done for different values of the rate of HGT h and for three different alignment kernels. As expected, the divergence is only marginally influenced by the HGT rate, but the saturating value of the divergence depends strongly on the choice of alignment kernel, with *mmseqs2* being able to merge sequences with average divergence higher than 10%. To find the relationship between the mutation rate μ and the average pairwise divergence of the sequences we peform a linear fit on data points for $\mu \leq 0.002$. This provides the conversion factor $\langle d \rangle \sim 20.8\,\mu$.

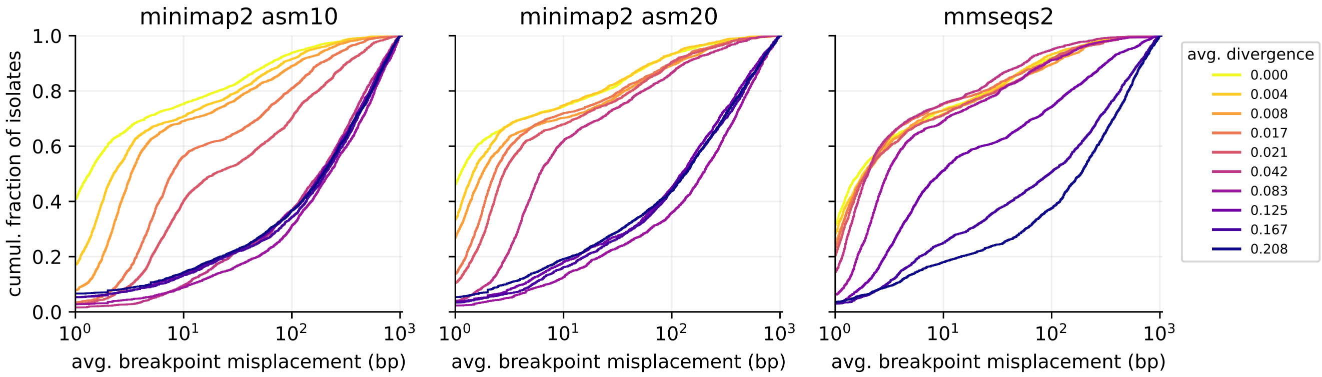

Fig. S 5 **Accuracy of PanGraph algorithm on synthetic data**. For each of our generated datasets we evaluate the average displacement of inferred pancontig breakpoints. The figure represents the cumulative distribution of average misplacements, stratified by the average pairwise divergence of sequences in pancontigs (see legend on the right). Each panel corresponds to a different alignment kernel used to infer the pangenome graph (see panel title). As divergence increases we observe a transition in the precision of breakpoint position estimation. At low divergence the position of most breakpoints is correctly estimated withing a few bps, while at high divergence most breakpoints can be misplaced by multiple hundreds of bps. The transition value for the divergence depends on the alignment kernel used, with *mmseqs2* having the highest accuracy at the cost of higher computational time.

V. BENCHMARK OF PANGRAPH ON REAL DATA

We benchmark *PanGraph* on real data, focusing on the computational performances and the properties of the resulting pangenome graphs.

A. Dataset selection

We downloaded sets of annotated and complete chromosomes from RefSeq (O'Leary *et al.*, 2016) for 5 different bacterial species: *Klebsiella pneumoniae* (KP), *Helicobacter pylori* (HP), *Prochlorococcus marinus* (PM), *Mycobacterium tuberculosis* (MT) and *Escherichia coli* (EC). In Table I in the main text we report some summary statistics for these datasets. The number of isolates per species ranges from several hundreds for EC to 10 for PM.
These data has been chosen as part of a PanX (Ding *et al.*, 2018) analysis dated March 2018.[4] PanX is a pipeline for the analysis of bacterial pangenome. The pipeline takes as input a set of annotated genomes, and it clusters and aligns homologous genes. We used the PanX analysis to estimate the dataset diversity.
From this analysis we evaluated the average pairwise sequence divergence $\langle d_{\text{core}} \rangle$ of core genes, the average genome length $\langle L_{\text{gen}} \rangle$, the total pangenome length L_{pang}, the total core genome length L_{core}, and the total soft-core genome length $L_{95\%}$, where soft-core genes are defined as genes being present in at least 95% of the isolates. These quantities are reported in Table I in the main text.[5]
EC is the dataset with the highest number of isolates (307) and is also the one with the biggest pangenome. MT is the species with the smallest sequence divergence ($\langle d_{\text{core}} \rangle \sim 0.03\%$) and the biggest core genome fraction. The two datasets with highest sequence divergence are HP ($\langle d_{\text{core}} \rangle \sim 4\%$) and PM ($\langle d_{\text{core}} \rangle \sim 24\%$). For HP the average sequence divergence sits on the limit of what *PanGraph* is able to accurately merge when using the *minimap2* kernel (cf. Fig. 5), but is merged correctly by the *mmseqs2* kernel. The sequence divergence of PM sits instead beyond the capabilities of all alignment kernels.

B. Pangenome graph construction

We built pangenome graphs using five different options for the alignment kernel:

- kernel *minimap2* with option *asm10* and default values for the energy parameters $\alpha = 100$, $\beta = 10$.
- kernel *minimap2* with option *asm20* and default values for the energy parameters $\alpha = 100$, $\beta = 10$.
- kernel *mmseqs2* and default values for the energy parameters $\alpha = 100$, $\beta = 10$.
- kernel *minimap2* with option *asm10* and null energy parameters $\alpha = 0$, $\beta = 0$.
- kernel *mmseqs2* and null energy parameters $\alpha = 0$, $\beta = 0$.

From the results described in the previous section (see Fig. 5) we expect *mmseqs2* to be able to merge sequences with higher divergence than *minimap2*. Moreover from the definition of the pseudo-energy eq. (1) in the main text, the value of the energy parameters β defines an upper threshold for sequence divergence $d \sim 1/\beta$. Homologous sequences with divergence higher than this value will result in positive pseudo-energy and will not be merged in a single pancontig. For $\beta = 10$ the critical divergence threshold is $d \sim 10\%$. Executing pangraph with the option $\alpha = 0$, $\beta = 0$ will result in the merging of more diverged sequences, at the cost of a more fragmented pangenome graph containing a higher number of shorter pancontigs.

C. Benchmark results

The results of the benchmark are displayed in Fig. 6. In each panel data are divided by species, with colors and texture identifying the five possible choices for the alignment kernel.
Execution time (cf. panel B) depends on the dataset size and ranges from few minutes for PM to several hours for EC. Aligning sequences with *mmseqs2* consistently requires more time than *minimap2*. The time required to build a pangenome graph for the 307 chromosomes of EC is only 5h for *minimap2*, and around 24h for *mmseqs2*. The latter also has a higher minimum memory requirement (panel C, around 8 Gb when running on 8 cores) but this is comparable to *minimap2* when aligning several tens of isolates.

---

[4] The list of accession numbers is available on the repository: `https://github.com/neherlab/pangraph/blob/master/script/config/accession_numbers.csv`. We report for completeness that two of the isolates from the *E. coli* dataset from this analysis, namely `NC_020518` and `NC_017663`, were subsequently removed from RefSeq. We verified that their removal from the dataset does not significantly impact core-genome and soft-core genome size, but decided to utilize the complete dataset as per original PanX analysis.

[5] L_{core} is the length of the alignment used to estimate sequence divergence $\langle d_{\text{core}} \rangle$. However this quantity is sensitive to annotation errors. This is particularly evident in the *E. coli* dataset, for which $L_{\text{core}} = 0.7$ Mbp is likely to be an underestimation of the real core genome size. $L_{95\%}$ is instead less sensitive to annotation errors and usually considered a better core-genome size estimate. For reference, the *E. coli* core genome is usually considered to be around 1500-2500 genes in size, depending on the definition used (Horsfield *et al.*, 2023; Park *et al.*, 2019; Sutton *et al.*, 2021).

We quantify the properties of pancontigs by looking at three main statistics: their total number (panel D) the size of the minimal set of pancontigs that includes at least 50% of all the graph sequence (L50 statistics, panel E) and the length of the shortest pancontig in this minimal set (N50 statistics, panel F). As expected, the use of null energy parameters results on average in a more fragmented graph, having more and shorter pancontigs. In general the pangenome graphs of our datasets have around 10^3 / 10^4 pancontigs, with 50% of the sequence being contained in around 10% of them, having N50 length of around 1-10 kbp.
The total pangenome length (panel G) depends both on the number of isolates and the ability of the alignment kernel to merge homologous sequences. When setting the energy parameters to zero more diverged sequences are merged into the same pancontigs and the total pangenome graph size is consistently smaller. The total graph size is in general much smaller than the cumulative size of the genomes it contains (panel I), with compression up to 1-2% of the total size for graphs with a high number of isolates (EC) or with small accessory genome (MT). The fraction of core sequence (panel H) is consistent with the species expectation, with MT having the highest core fraction.

Two species in our datasets hit the limits of the capabilities of *PanGraph*: HP and PM.
HP contains some homologous sequences with divergence higher than the default threshold $d = 1/\beta \sim 10\%$. Removing this threshold by using null energy parameters results in a graph with fewer pancontigs, smaller L50, greater fraction of core pangenome and higher level of sequence compression. These are all indications of more complete merging of homologous sequences. This effect is more marked for *mmseqs2* than for *minimap2*, in agreement with their different divergence limit (cf. Fig. 5). The HP datasets represents an example in which relaxing the default divergence threshold of pangraph and using a more accurate alignment kernel results in a more consistent pangenome graph.
Conversely, the PM dataset contains highly diverged isolates, whose divergence go beyond the capabilities of both alignment kernels ($\langle d_{\text{core}} \rangle \sim 27\%$, cf. Table I in the main text). Only *mmseqs2* manages to partially merge some of the least diverged core sequences. This results in a small core genome fraction and poor sequence compression. In its current state *PanGraph* cannot meaningfully merge genomes with such high divergence into a pangenome graph.

VI. GRAPH MARGINALIZATION

Given a pangenome graph containing many different genomes, *PanGraph* is capable of marginalizing it on a subset of strains with the `marginalize` command. Marginalization removes undesired paths and merges any remaining transitive pancontigs, i.e. pancontigs that are co-linear in all of the remaining paths. The resulting graph should be equivalent to the one obtained by directly building a graph on the selected subset of strains, with the added advantage that marginalization is a fast operation. This makes it more convenient to build a pangenome graph for the full set of isolates considered, and then marginalizing it multiple times on interesting subsets, rather than building a new graph for each subset.

We used genomes from the dataset described in the previous section to verify that marginalized graphs are compatible with graphs built directly from subset of strains. For each species we selected 50 different isolates (10 for PM) and built pangenome graphs using standard parameter values and the *minimap2* kernel with *asm20* option. For each species we then randomly picked 50 different pairs of isolates (all 45 for PM).[6] For each pair we then built pairwise graphs using the same parameters, and compared them to the corresponding marginalized graph, obtained by projecting the full pangenome graph for the 50 isolates on the selected pair (cf. Fig. 6A, main text).
The comparison was performed by considering that each graph partitions a genome into pancontigs. Each pancontig can either be shared with the other member of the pair, or can be private to the isolate (cf. Fig. 6A, main text). For each isolate we consider the two partitions defined by the marginalized and pairwise graphs, and generate their intersection. Each block in this intersection can belong to two different categories, depending on whether the two partitions agree or disagree on whether the block is shared. Moreover, we can separate blocks on which we find agreement in two further categories: blocks that are shared and blocks that are private on both graphs.
For each pair of genomes and each type of block we evaluate the fraction of the genome that they cover and their average size. In Fig. 7 we display the distribution of these quantities across all pairs for the different species considered and the block types described above.
In all species except HP and PM we find very good agreement between pairwise and marginalized graphs: segments on which the graphs *disagree* represent only a very minor fraction of the genome (much smaller than the fraction of shared

[6] The list of selected pairs for each species is available on the repository: `https://github.com/neherlab/pangraph/blob/master/script/config/marginalize_pairs.csv`

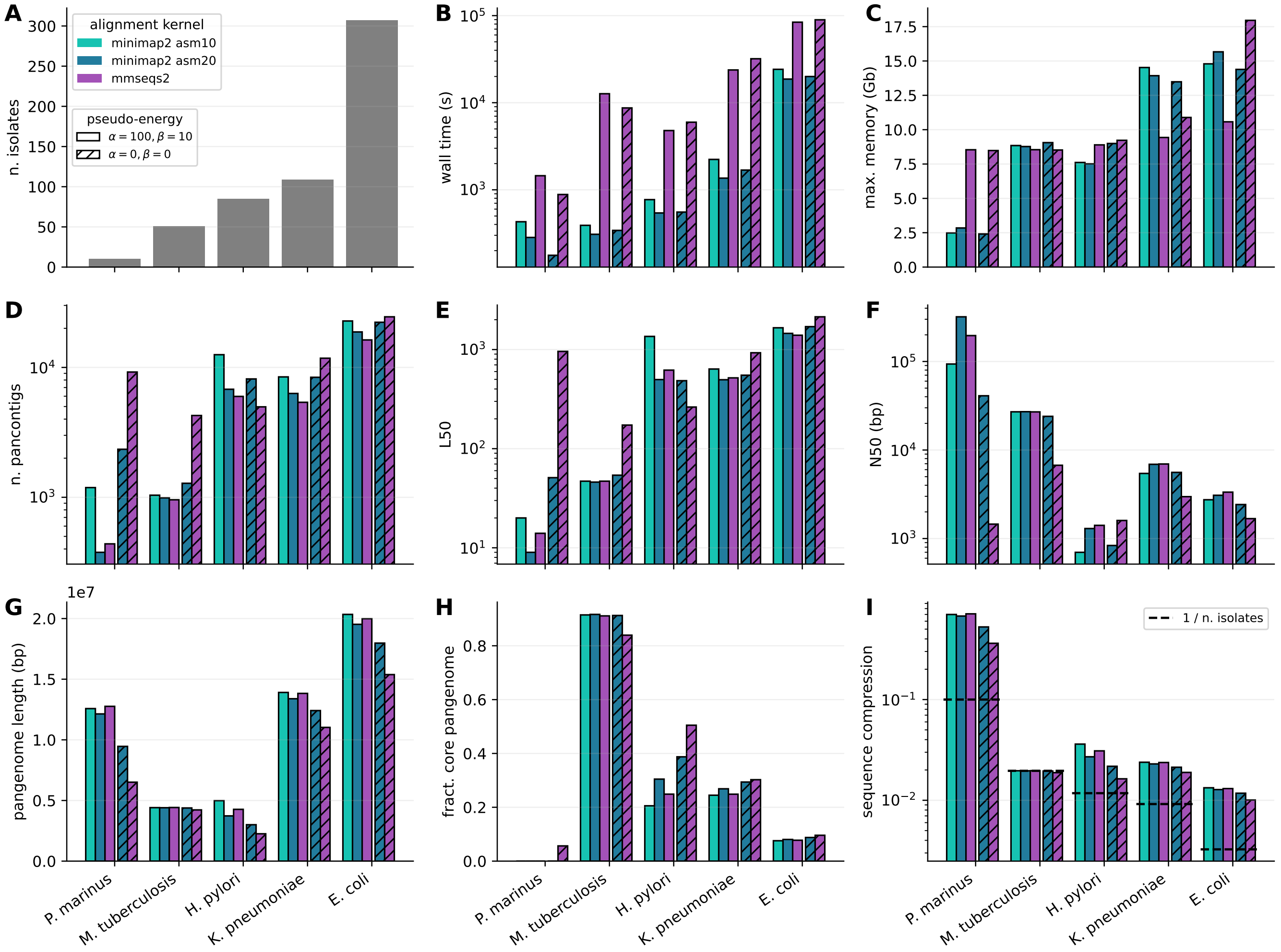

Fig. S 6 **Benchmark on real data**. We run *PanGraph* on datasets consisting of 5 different bacterial species, and using 5 different options for the alignment kernel as described in the text. Runs were performed using 8 cores. **A**: number of isolates for each species. **B**: algorithm wall-time. **C**: maximum memory usage. **D**: total number of pancontigs in the pangenome graph. **E**: minimum number of pancontigs that can add up to 50% of the total pangenome graph length. **F**: threshold length such that the cumulative size of all pancontigs longer than this threshold is more than 50% of the full pangenome graph. **G**: total length of the pangenome graph (sum of all pancontig lengths). **H**: fraction of the pangenome graph that is composed of core pancontigs, i.e. pancontigs that occur exactly once per isolate. **I**: ratio between the length of the pangenome graph and the total length of all genomes included in the graph. Since maximal compression also depends on the number of isolates N, we mark with horizontal lines the value of $1/N$ for each species.

or private blocks), and have size compatible with the default pancontig length threshold of *PanGraph* (L_{min} =100 bp). As described in the previous section, the divergence of the HP dataset sits on the limit of what the *minimap2* alignment kernel can successfully handle. As such, the results of merging different graphs together can depend on the order of merging, and pairwise and marginalized graphs can potentially show some disagreement. In this case the disagreement remains minor and only extends to few percents of the genome size, with disagreement blocks having size only slightly bigger than the 100 bp threshold of *PanGraph*. PM represents instead an example of a dataset with divergence much beyond the limit of the alignment kernel ($\langle d_{\mathrm{core}} \rangle \sim 27\%$). As a consequence, highly diverged homologous sequences are not merged, and the great majority of segments are private and have large size. Results also show a strong order-dependence, with some *disagreement* blocks having size of 10s of kbp.

REFERENCES

Ding, W., F. Baumdicker, and R. A. Neher (2018), Nucleic acids research **46** (1), e5.

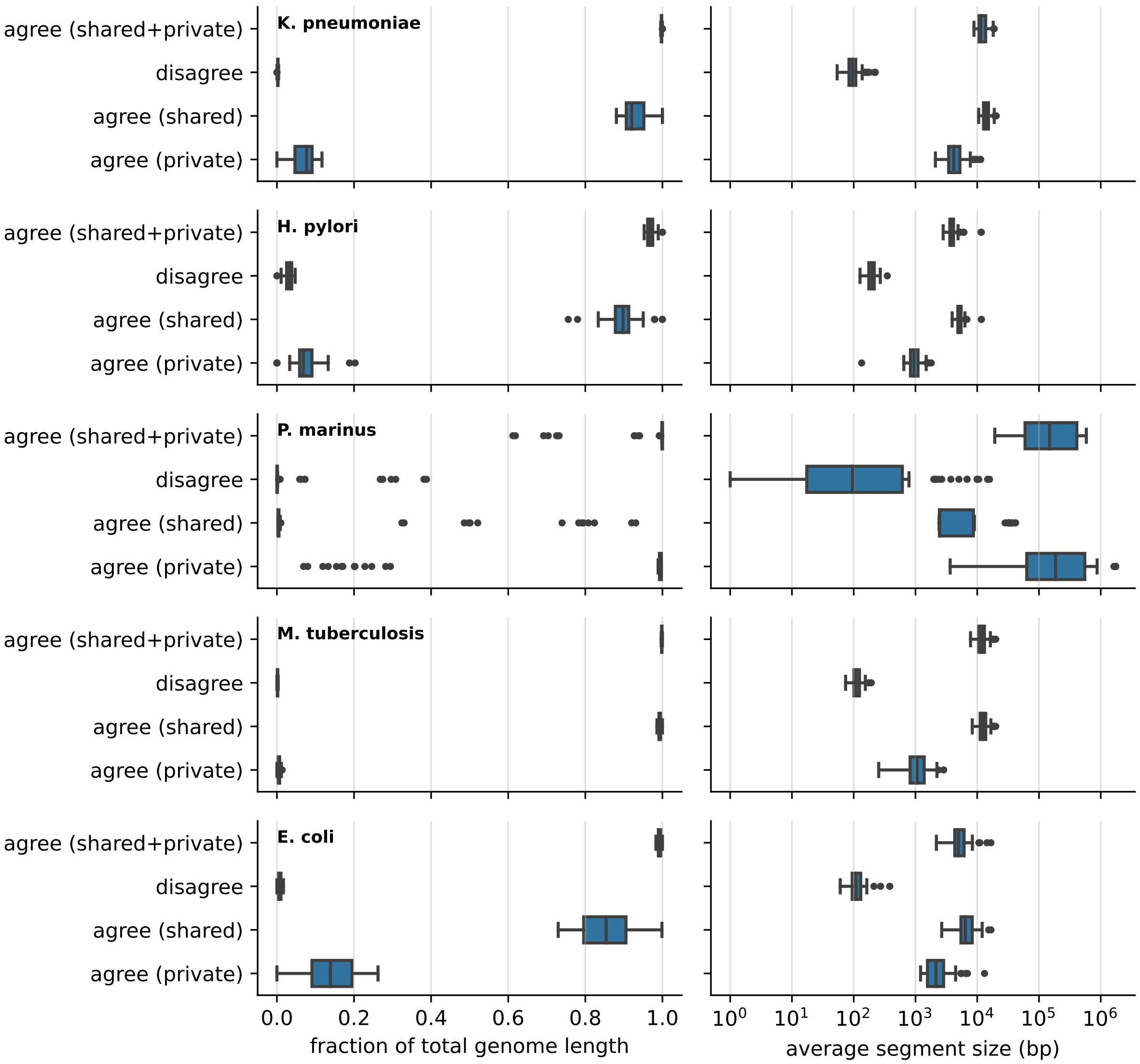

Fig. S 7 **Marginalized graphs are compatible with pairwise graphs**. We compare marginalized graphs with pairwise built graphs for different random pairs of strains from 5 different species, as described in the text. Each graph defines a partition of a genome into segments, that can either be shared in the pair or private to the genome. We intersect these two partitions, and categorize segments based on whether the two partitions *agree* or *disagree* on their sharing. Agreement segments are further split depending on whether the segment is *shared* or *private* on both graphs. The figure displays the distribution of the average size of these segments (right column) and the average fraction of genome that they cover (left column) for the 50 different pairs considered (45 for PM). For all species with sequence divergence compatible with the limit of the alignment kernel used (KP, MT, EC), we find very good agreement between marginalized and pairwise graphs, with disagreement segment only covering a very small fraction of the genome and having size compatible with *PanGraph* threshold pancontig length (100 bp).

Horsfield, S. T., N. J. Croucher, and J. A. Lees (2023), bioRxiv , 2023.

Hudson, R. R. (2002), Bioinformatics **18** (2), 337.

Katoh, K., K. Misawa, K.-i. Kuma, and T. Miyata (2002), Nucleic acids research **30** (14), 3059.

Kuhn, H. W. (1955), Naval research logistics quarterly **2** (1-2), 83.

O'Leary, N. A., M. W. Wright, J. R. Brister, S. Ciufo, D. Haddad, R. McVeigh, B. Rajput, B. Robbertse, B. Smith-White, D. Ako-Adjei, *et al.* (2016), Nucleic acids research **44** (D1), D733.

Park, S.-C., K. Lee, Y. O. Kim, S. Won, and J. Chun (2019), Frontiers in microbiology **10**, 834.

Sutton, G., G. B. Fogel, B. Abramson, B. Lauren, M. Todd, E. S. Liu, and T. Sterling (2021), F1000Research **10**.
